# Exploring a lead-free organic–inorganic semiconducting hybrid with above-room-temperature dielectric phase transition†

**DOI:** 10.1039/c9ra09289g

**Published:** 2020-05-05

**Authors:** Yuyin Wang, Shiguo Han, Xitao Liu, Zhenyue Wu, Zhihua Sun, Dhananjay Dey, Yaobin Li, Junhua Luo

**Affiliations:** State Key Laboratory of Structural Chemistry, Fujian Institute of Research on the Structure of Matter, Chinese Academy of Sciences Fuzhou Fujian 350002 P. R. China jhluo@fjirsm.ac.cn sunzhihua@fjirsm.ac.cn xtliu@fjirsm.ac.cn; University of Chinese Academy of Sciences, Chinese Academy of Sciences Beijing 100039 P. R. China; State Key Laboratory of Crystal Materials, Shandong University Jinan 250100 China

## Abstract

Recently, organic–inorganic hybrid lead halide perovskites have attracted great attention for optoelectronic applications, such as light-emitting diodes, photovoltaics and optoelectronics. Meanwhile, the flexible organic components of these compounds give rise to a large variety of important functions, such as dielectric phase transitions. However, those containing Pb are harmful to the environment in vast quantities. Herein, a lead-free organic–inorganic hybrid, (C_6_H_14_N)_2_BiCl_5_ (CHA; C_6_H_14_N^+^ is cyclohexylaminium), has been successfully developed. As expected, CHA exhibits an above-room-temperature solid phase transition at 325 K (*T*_c_), which was confirmed by the differential scanning calorimetry measurement and variable temperature single crystal X-ray diffraction analyses. Further analyses indicate the phase transition is mainly governed by the order–disorder transformation of organic cyclohexylaminium cations. Interestingly, during the process of phase transition, the dielectric constant (*ε*′) of CHA shows an obvious step-like anomaly, which displays a low dielectric constant state below *T*_c_ and a high dielectric constant state above *T*_c_. Furthermore, variable temperature conductivity combined with theoretical calculations demonstrate the notable semiconducting feature of CHA. It is believed that our work will provide useful strategies for exploring lead-free organic–inorganic semiconducting hybrid materials with above room temperature dielectric phase transitions.

## Abstract 2

Recently, organic–inorganic hybrid lead halide perovskites have attracted great attention for optoelectronic applications, such as light-emitting diodes, photovoltaics and optoelectronics.

## Graphical abstract

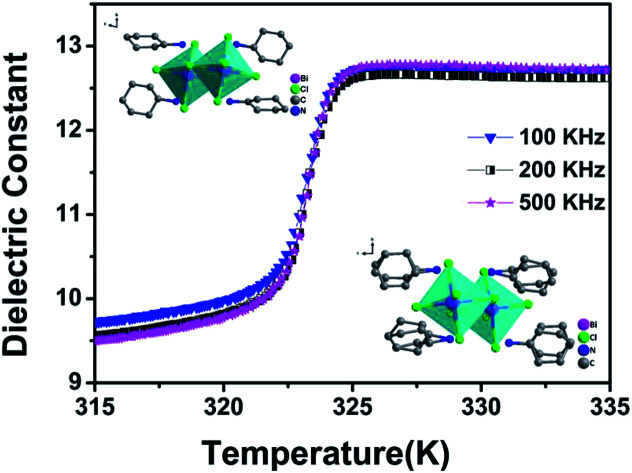

## Introduction

Solid state switchable dielectric materials have attracted great attention due to their significant roles in signal processing, phase shifters, data communication, varactors and sensors, *etc.*^1–8^ It is known that the dielectric constant reflects the electric polarizability of a material, which relates to the dipole movement such as molecular rotation. Polar molecules in the solid state usually show smaller electric permittivities than in the liquid state because of the “freezing” and “melting” of the molecular reorientations, which respectively corresponds to a low temperature phase (LTP) and a high temperature phase (HTP), which makes phase transition materials promising candidates as new dielectric switches. In addition, in consideration of practical applications, the optimal phase transition temperature range of dielectric switching materials is between 290 K and 365 K, particularly at room temperature, which can benefit from changes in the environment.^9^ On the other hand, an economical and environmentally friendly solution is also required for a wide range of applications.^9,10^ Therefore, it is of great significance to explore above room-temperature lead free dielectric phase transition materials.

Organic–inorganic hybrid materials have attracted significant attention owing to their diverse properties, such as phase transitions and semiconducting properties.^11–15^ Switchable dielectric materials have been found in some organic–inorganic phase transition hybrid. Meanwhile, it has been proved that cyclic organic amines is promising candidates to design dielectric phase transition materials due to their flexible structures. As a result, some dielectric phase transition hybrid have been developed using 3-, 5-, 6- and 7-heterocyclic organic amines.^15–22^ For example, bis(cyclohexylaminium) tetrabromo lead, a layered hybrid compound, which has excellent dielectric switching performance, that is, the dielectric constant values vary from 25 to 400 at different temperature, which features an excellent property of dielectric switching.^23^ Recently reported (hexamethyleneimine)PbBr_3_, a one-dimensional ABX_3_-type perovskite-like hybrid, also exhibits a phase transition with high dielectric switching property.^24^ However, these Pb containing materials are harmful to the environment in their vast quantities of use.^25–28^ One of the most effective approaches is to move away environmentally harmful element lead.^29,30^ As same as Pb^2+^ ions electron configurations (6s^2^6p^0^), Bi^3+^ ions are low toxicity to the environment, which also can form a variety of organic–inorganic compound.^31^ Therefore, it is expected as an environmentally friendly alternative for lead containing materials.

Herein, we present an above-room-temperature lead-free organic–inorganic hybrid material, (C_6_H_14_N)_2_BiCl_5_ (CHA; C_6_H_14_N^+^ is cyclohexylaminium), in which the corner-sharing BiCl_6_ octahedral clusters construct a zero-dimensional perovskite-like anionic framework. CHA shows remarkable dielectric responses that can be switched by phase transition at ∼325 K (*T*_c_). The mechanism is the order–disorder transformation of organic cyclohexylaminium cation. The order–disorder of organic cations accounts for its switchable dielectric activities. Therefore, CHA is considered as a dielectric switch owing to its phase transition. Moreover, CHA displays notable semiconducting properties with an optical bandgap of ∼3.20 eV. Thus, it is expected that our work will provide useful strategies for exploring organic–inorganic semiconducting hybrid materials with the above room temperature dielectric phase transitions.

## Experimental section

### Materials and methods

#### Synthesis

None of the chemicals required for the analytical stage were further purified for synthesis. CHA was synthesized by the reaction of cyclohexylamine and Bi_2_O_3_ with hydrochloric acid. Firstly, the BiCl_3_ was synthesized by the reaction of bismuth oxide (Bi_2_O_3_) (1 g) with excessive hydrochloric acid in a bath. Secondly, an appropriate amount of cyclohexylamine (0.43 g) was added into the above solution (the stoichiometric molar ratio of cyclohexylamine and bismuth(iii) with a 2 : 1 molar ratio) after stirring for 20 minutes. Then, the mixture was heated for 20 minutes until the precipitate dissolved completely at 373 K. At room temperature, white crystals grow slowly over several days from an aqueous solution (ESI, Fig. S1†). By cooling method at a cooling rate of 0.5 °C d^−1^ from hot solution of 373 K, large size white crystals were grown in the above clarified solution.

#### Thermal measurements

DSC analyse was carried out on a NETZSCH DSC 200 F3 with the temperature range of 290–350 K. CHA crystal was put on aluminum crucibles under a nitrogen atmosphere. The cooling or heating rates was 10 K min^−1^. A Netzsch STA 449C device was used for thermogravimetry (TG) analysis, with a temperature range of 285–1000 K and a heating rate of 10 K min^−1^.

#### Dielectric measurements

The CHA a powder-pressed pellet was covered by silver conductive paste were subjected to dielectric experiments. For CHA, the temperature dependence of the dielectric constants was performed on the TH2828A impedance analyzer with a temperature range of 315–335 K. The frequencies of 100 KHz, 200 KHz, 500 KHz and 1 MHz were recorded by the complex dielectric constant (*ε* = *ε*′ − i*ε*′′, where *ε*′′ is the imaginary part) of the real part on the conditions of temperature dependence.

#### Single-crystal X-ray diffraction

Single crystal X-ray diffraction analyses were measured from the Bruker D8 Quest/Venture diffractometer by using Mo Ka radiation (*λ* = 0.77 A) at 333 K and 200 K.

### Powder X-ray diffraction

Mini Flex II powder X-ray diffraction (PXRD) analysis was performed to determine the purity of CHA with the 2*θ* range of 5–50° and a step size is 0.02°. The theoretical simulation simulated from single-crystal X-ray diffraction structure of CHA agrees well with the experiment PXRD (ESI, Fig. S2†).

## Results and discussion

**Fig. 1:**
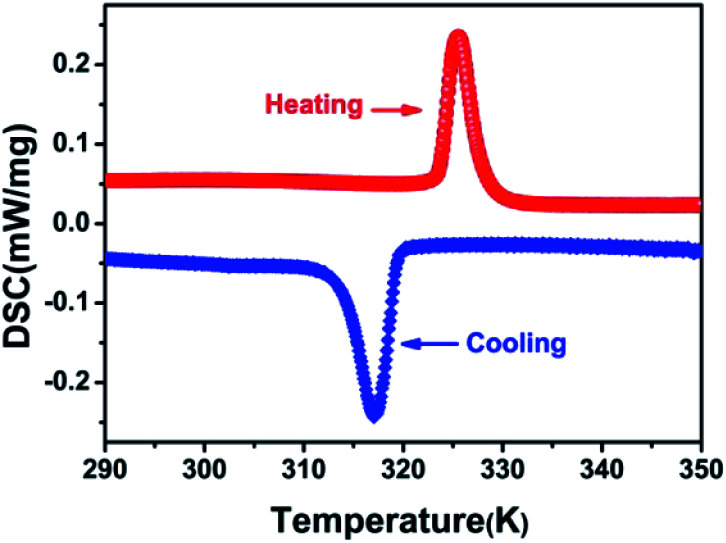
DSC curves of CHA with rate of 10 K min^−1^ in a heating–cooling cycle.

**Fig. 2:**
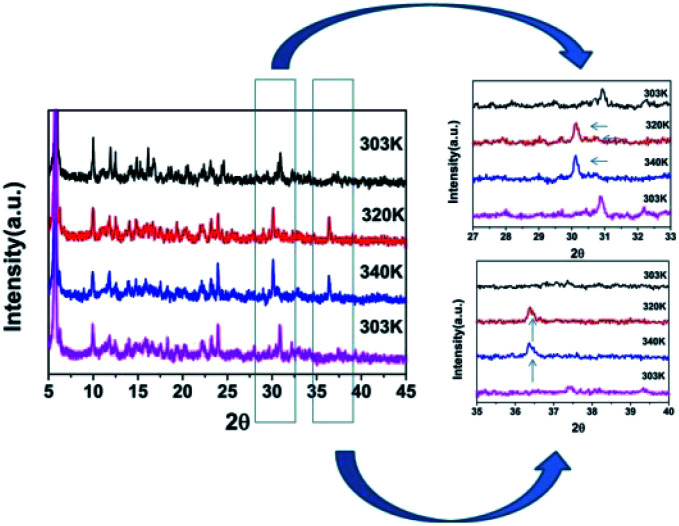
Variable-temperature PXRD patterns of CHA.

DSC can be used to confirm phase transition of a solid material.^32^ From DSC curve of CHA (Fig. 1), an exothermic peak (317 K) and an endothermic (325 K) peak were observed upon cooling and heating, respectively, which revealed that it is a reversible phase transition. The large heat hysteresis of 8 K is indicative of the discontinues first-order one.^33^ Fig. S3† represents the thermogravimetric analysis (TGA) curves for CHA. It reveals that it has thermal stability until 520 K without any thermal decomposition, indicating the excellent phase stability. In order to further investigate on phase transition of CHA, as illustrated in Fig. 2, variable-temperature PXRD of well-ground crystal powder was performed at 303 K, 320 K, 340 K and back to 303 K, respectively. It is noticed that the PXRD patterns remain unchanged at room temperature (303 K). Meanwhile, the PXRD patterns at high temperature present obvious changes, which can been observed in comparison with that at room temperature. For example, the diffraction peaks at 31° display movements towards low-angle. At the same time, a new peak arrears in the 36.5°, which also confirms the phase transition of CHA.

**Fig. 3:**
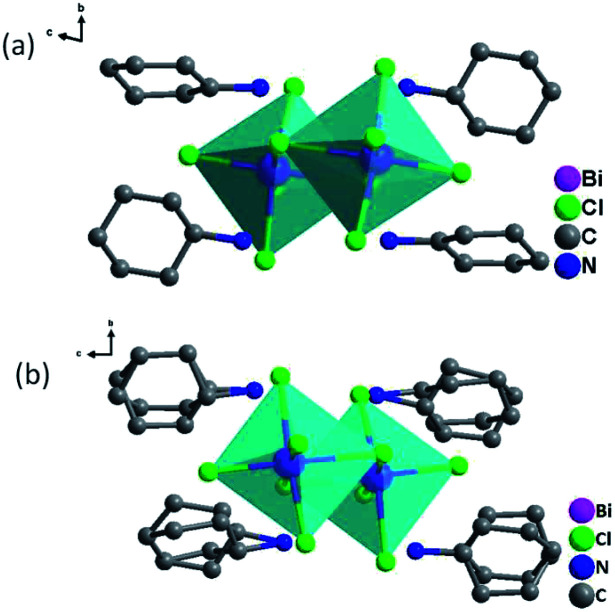
(a) Asymmetric unit of CHA at 200 K; (b) asymmetric unit of CHA at 333 K.

As shown in Fig. 3 and S4,† the basic unit of CHA consists of a discrete inorganic binuclear cluster, [Bi_2_Cl_10_]^4−^ and four equivalent organic cyclohexylaminium cations. The central Bi^3+^ is coordinated by six chlorine atoms as a bridging agent, forming a zero-dimensional structure in space. Interestingly, the [Bi_2_Cl_10_]^4−^ octahedral shape becomes a slightly distorted configuration. In order to better understand the structural transformation, the crystal structure was revealed under the conditions of 333 K (HTP) and 200 K (LTP) by means of variable temperature X-ray diffraction (Table S1†). The crystal structures of HTP and LTP are obviously different, which proves the existence of phase transition. In case of LTP structure, the space group is *P*1̄, and the cell parameters are *a* = 11.9718(4) Å, *b* = 12.4092(4) Å, *c* = 15.8390(6) Å, *α* = 76.4740(10)°, *β* = 69.2450(10)°, *γ* = 89.3230(10)°, *Z* = 2, *V* = 2132.77 Å^3^. It contains the basic unit of a discrete inorganic dual-core [Bi_2_Cl_10_]^4−^ clusters and four equivalent of cationic organic cyclohexylamine. Interestingly, the negative ion framework is composed of surface-shared octahedra in which the central Bi atoms are coordinated by six iodine atoms that act as bridging agents (Fig. 3). And the cationic and anionic moieties are ordered in the LTP.

The HTP structure is in a monoclinic system with the space group *P*2_1_/*c*, and cell parameters of *a* = 11.9592(3) Å, *b* = 12.7479(2) Å, *c* = 31.1058(6) Å, *β* = 108.8270(10)°, *Z* = 4 and *V* = 4488.5 Å^3^. From LTP to HTP, the volume of the crystal cell was significantly doubled. The change of the crystal cell parameter confirmed that the crystal cell had a phase change in CHA, which matches well with the DSC measurement. The CHA phase change was confirmed from another perspective. The difference in the high-temperature phase is that organic cation disordered in high temperature phase due to thermal effect. While the protonated cyclohexylamine cation becomes highly disordered, and the pentachlorobium negative ion skeleton remains in an ordered fashion. As shown in Fig. S5b,† cyclohexylamine cations are strictly disordered, and all atoms adopt two equivalent disordered positions. Compared with the variable temperature structure of CHA, the most significant structure feature of CHA is that the inorganic skeleton remains ordered (Fig. S4†), while the protonated cyclohexylaminium cation becomes highly disordered with the crystal point group changes from *P*1̄ to *P*2_1_/*c*. As shown in Fig. S6,† from the viewpoint of symmetry breaking, as the temperature increases, the crystallographic symmetry of CHA transformed from the 1̄ to 2/*m* point group.

**Fig. 4:**
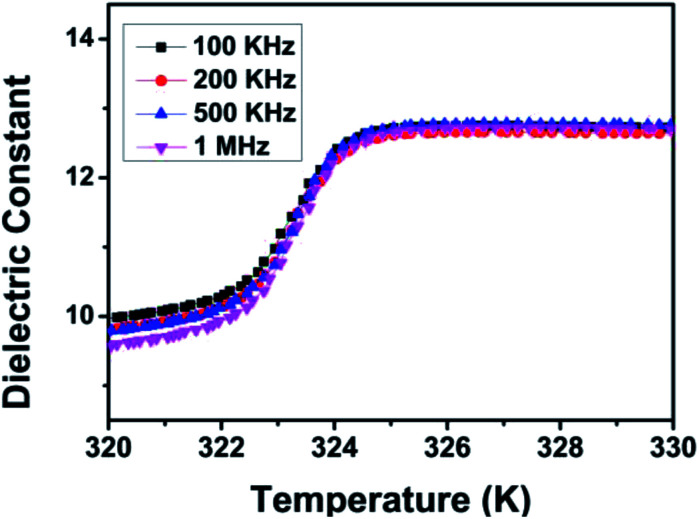
Temperature-dependent dielectric constant of CHA at different frequencies.

Dielectric response, usually apparently unusual at the phase transformation point, is one of the important indices for characterization of structure transformation. The dielectric constant were measured at four fixed frequencies (100 K, 200 K, 500 K and 1 M) in the temperature range of 315 to 335 K. With increasing temperature, the dielectric constant *ε*′ shows a relatively large step-like transition at about 325 K, consistent with the results of variable temperature PXRD and thermal analysis discussed above (Fig. 4). HTP has a higher dielectric constant, while LTP has a lower dielectric constant. The dielectric constant exhibits strong temperature dependence. In other words, dielectric constant increases gradually on heating, which reaches a maximum value near the *T*_c_. Then the dielectric constant becomes stationary. That is, the dielectric constant *ε*′ of CHA exhibits a marked increase at temperatures below the 325 K (*T*_c_), followed by a significant step-like at *T*_c_, where the value of epsilon is moved rapidly from 9.5 in the vicinity of the *T*_c_ to 12.5. The change of dielectric behavior with temperature is obviously consistent with the properties of switchable molecules. The intense dielectric anomaly in the *T*_c_ region triggered by this temperature brings to mind the typical characteristics of the switchable dielectric material. This switch medium is considered to be due to order–disorder behavior of the cyclohexylaminium cation. These results indicate that CHA is a promising above-room-temperature switchable dielectric material.

**Fig. 5:**
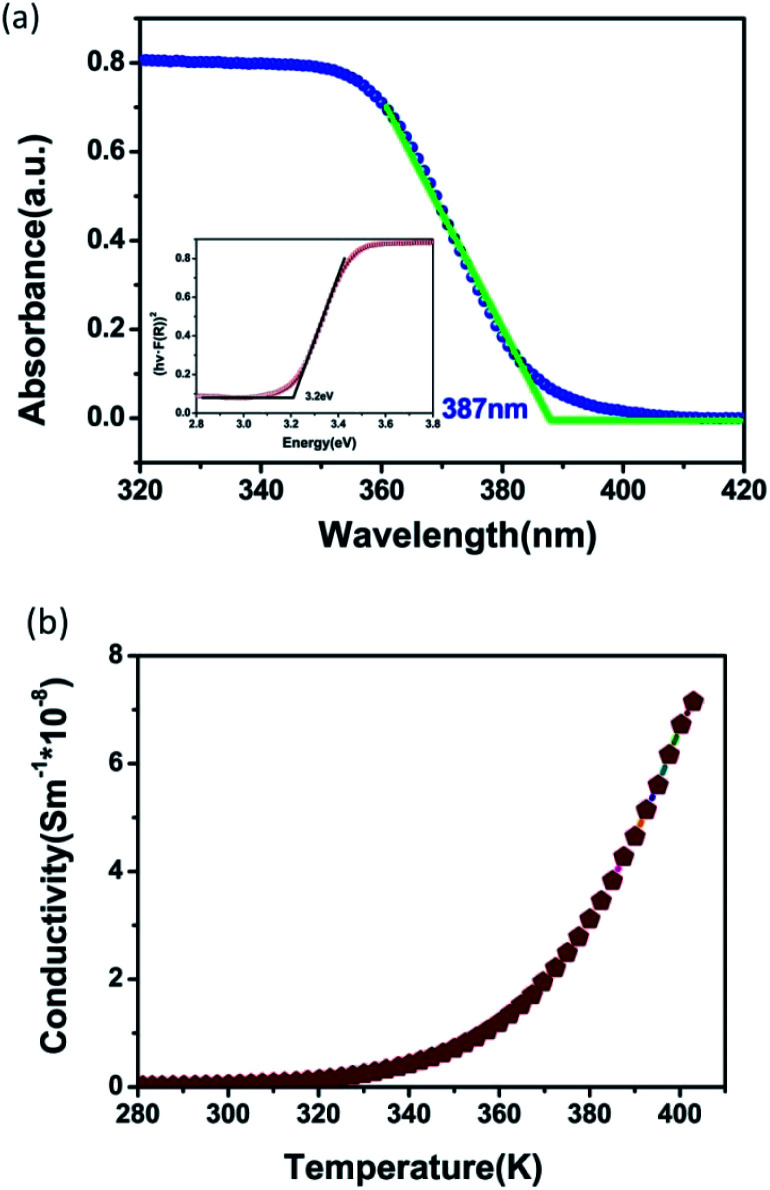
(a) UV-vis spectroscopy of CHA; inset is the calculated bandgap; (b) temperature dependence of the a.c. conductivity of CHA.

We performed Ultraviolet-visible (UV-vis) spectroscopy to explore the optical bandgap (E_g_). As shown in Fig. 5a, the crystals of CHA show that the absorption edge is about 387 nm. Based on diffuse reflection spectrum, the estimated optical band gap of CHA crystals is about 3.20 eV, which can be calculated from the Tauc plot^34^ (inset of Fig. 5a) of [*hνF*(*R*)]^1/*n*^*versus* the energy (eV), where linear region was extrapolated to the *x*-axis intercept. Variable-temperature conductivity was performed to further understand the semiconducting feature of CHA, and Fig. 5b reveals that the positive slopes of CHA increase gradually upon increasing temperature, which proves that CHA is semiconducting material.

**Fig. 6:**
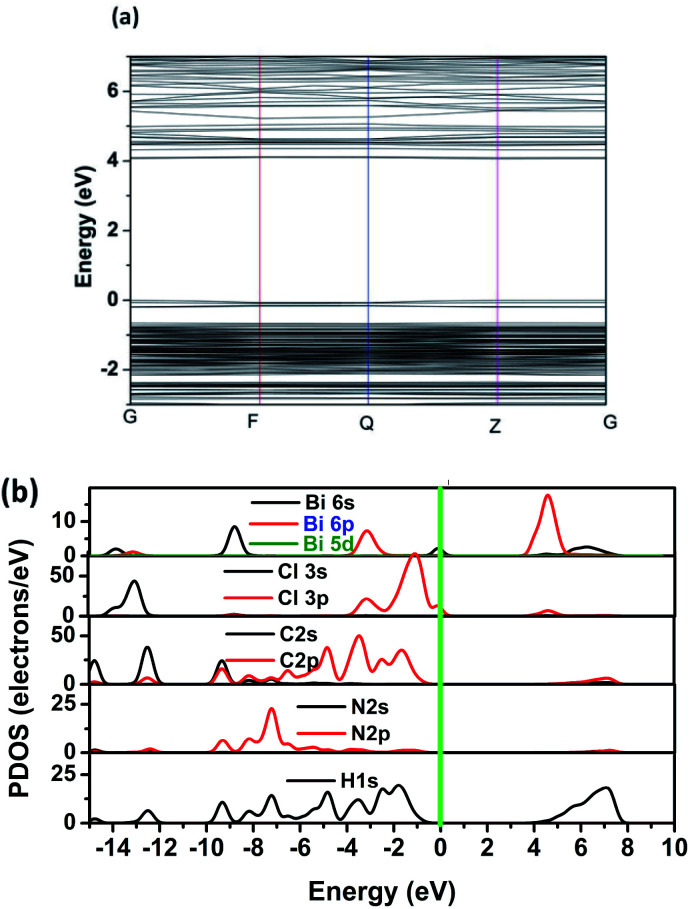
(a) The calculated band structure of CHA. (b) The partial density of states (PDOS) of CHA.

To obtain further discernment of the electronic properties, the electron energy band structure and partial state density (PDOS) are calculated by means of density functional theory. Fig. 6a displays the calculated band gap is 4.00 eV, which is slightly larger than the experimental one. The slightly difference in bandgap are mainly due to the limitations of DFT calculation.^35,36^ In addition, the conduction band minimum is localized at the *Z* points, and the valence band maximum is localized at the *Z* point, which reveals that CHA is direct bandgap material with semiconducting characteristic. Partial density of states (PDOS) illustrate that the valence band maximum is mainly derived from CI 3p states, while the conduction band minimum mostly attributed to Bi 6p and Cl 3p states.

## Conclusions

In conclusion, we have presented a lead-free organic inorganic hybrid, CHA, which features a dielectric phase transition at 325 K. The phase transition was confirmed by dielectric measurements, DSC and variable temperature single-crystal structural analyses. Structural analysis shows that the order–disorder dynamics of organic moieties accounts for its phase transition. Moreover, the obvious step-like dielectric pattern indicates that CHA can be considered as switchable dielectric material. Interestingly, CHA also exhibits a semiconducting feature. This successful example may provide a new approach to design new lead-free dielectric phase transition materials.

## Conflicts of interest

There are no conflicts to declare.

## Supplementary Material

RA-010-C9RA09289G-s001

RA-010-C9RA09289G-s002
