# Supplementary material for: Exploring a lead-free organic–inorganic semiconducting hybrid with above-room-temperature dielectric phase transition
Source: RSC Adv. 2020 May 5;10(30):17492–6. doi: 10.1039/c9ra09289g (PMC9053409; doi:10.1039/c9ra09289g)
Supplement: RA-010-C9RA09289G-s001 [file RA-010-C9RA09289G-s001.pdf]

Electronic Supplementary Material (ESI) for RSC Advances.
This journal is © The Royal Society of Chemistry 2019

Supporting Information for

Exploring a Lead-free Organic-Inorganic Semiconducting Hybrid with Above-Room-Temperature Dielectric Phase Transition

Yuyin Wang,[ab] Shiguo Han,[ab] Xitao Liu,[ac*] Zhenyue Wu,[ab] Zhihua Sun,[a*] Dhananjay Dey,[a] Yaobin Li,[a] and Junhua Luo[a*]

[a]*State Key Laboratory of Structural Chemistry, Fujian Institute of Research on the Structure of Matter, Chinese Academy of Sciences, Fuzhou, Fujian 350002, P.R.China*

[b]*University of Chinese Academy of Sciences, Chinese Academy of Sciences, Beijing 100039,P.R. China*

[c]*State Key Laboratory of Crystal Materials, Shandong University, Jinan, 250100, China*

**To whom correspondence should be addressed. E-mail:*
jhluo@fjirsm.ac.cn；sunzhihua@fjirsm.ac.cn; xtliu@fjirsm.ac.cn

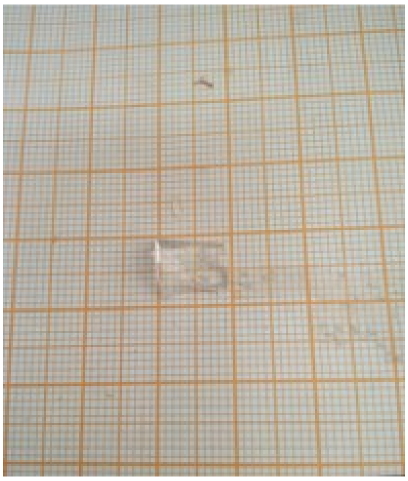

Figure. S1 Bulk crystal of **CHA**.

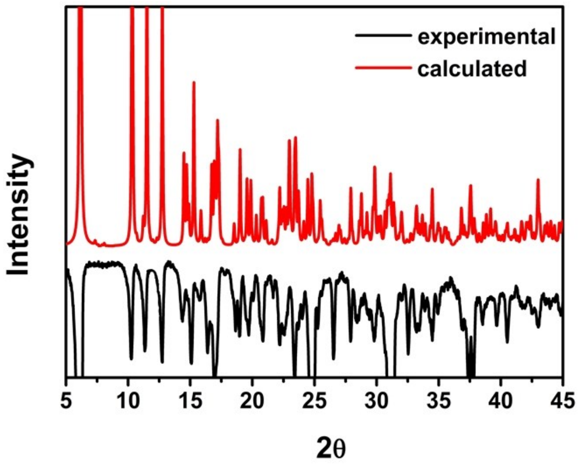

Figure S2. Powder X-ray Diffraction (PXRD) patterns of experimental data and the calculated data

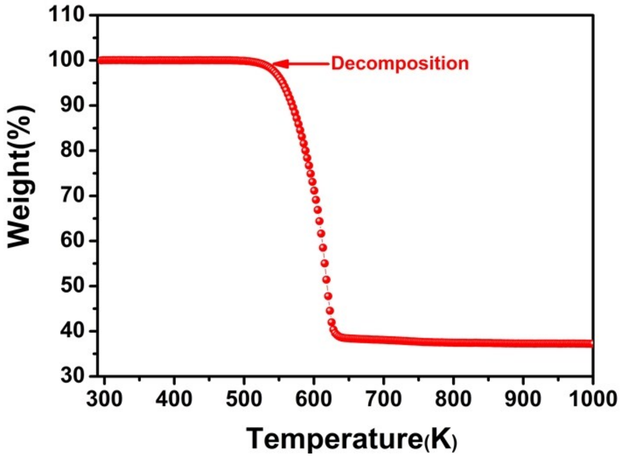

Figure S3. TG curves for **CHA**.

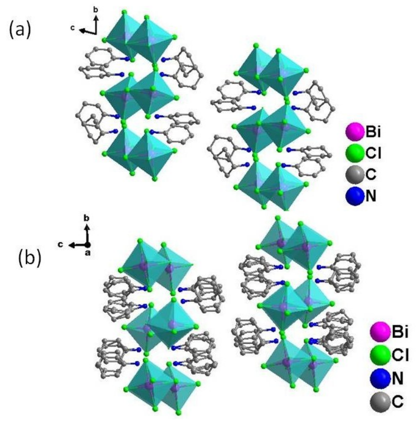

Figure S4. (a) Packing view of the unit cell at 200 K; (b) Packing view of the unit cell at 333K.

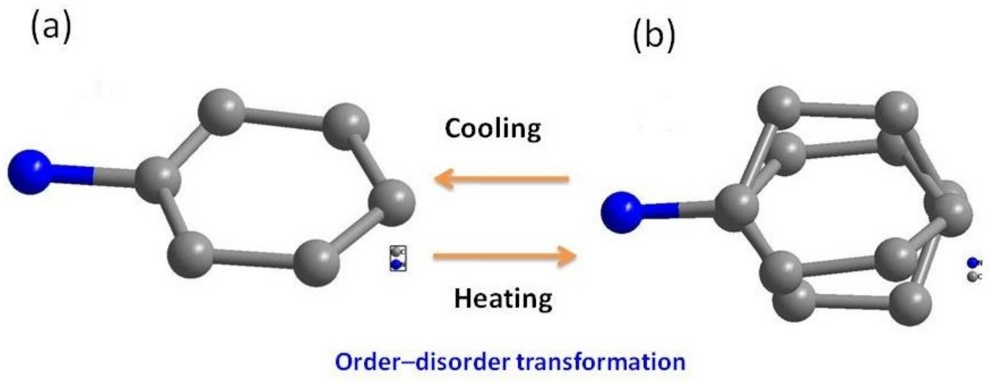

Figure S5. Order–disorder transformation of the cation in **CHA** during phase transition. cation attains (a) order state in RTP, and (b) disordered in HTP.

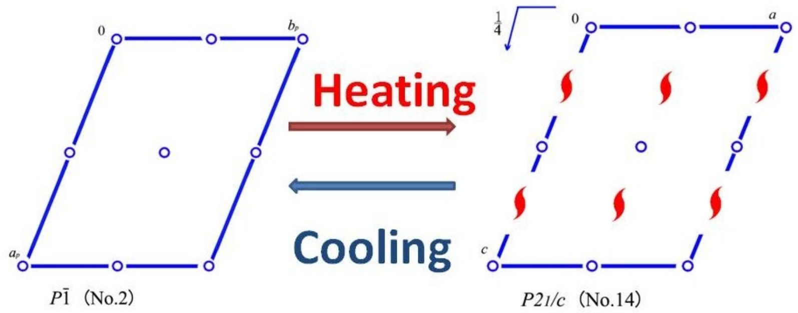

Figure S6. Symmetry transformation of CHA during the phase transition.

Table S1: Crystal structure and refinement detail of **CHA** at different temperatures.

Empirical formula	$C_{24}H_{56}N_4Cl_{10}Bi_2$	$C_{24}H_{56}Bi_2Cl_{10}N_4$
Formula weight	1173.19	1173.18
Temperature/K	200.15	333
Crystal system	triclinic	monoclinic

Space group	P-1	$P2_1/c$
a/Å	11.9718(4)	11.9592(3)
b/Å	12.4092(4)	12.7479(2)
c/Å	15.8390(6)	31.1058(6)
α/°	76.4740(10)	90
β/°	69.2450(10)	108.8270(10)
γ/°	89.3230(10)	90
Volume/$Å^3$	2132.77(13)	4488.50(16)
Z	2	4
$\rho_{calc}g/cm^3$	1.827	1.736
μ/mm^{-1}	8.887	8.446
F(000)	1128.0	2256.0
Radiation	MoKα (λ = 0.71073)	MoKα (λ = 0.71073)
2Θ range for data collection/°	4.78 to 55.14	4.812 to 55.008
Index ranges	-15 ≤ h ≤ 15, -16 ≤ k ≤ 16, -20 ≤ l ≤ 20	-15 ≤ h ≤ 15, -16 ≤ k ≤ 16, -40 ≤ l ≤ 40
Reflections collected	43425	92899
Independent reflections	9781 [R_{int} = 0.0643, R_{sigma} = 0.0507]	10303 [R_{int} = 0.0816, R_{sigma} = 0.0427]
Data/restraints/parameters	9781/175/365	10303/347/541
Goodness-of-fit on F^2	1.047	1.038
Final R indexes [I>=2σ (I)]	R_1 = 0.0520, wR_2 = 0.1444	R_1 = 0.0400, wR_2 = 0.0849
Final R indexes [all data]	R_1 = 0.0671, wR_2 = 0.1556	R_1 = 0.0781, wR_2 = 0.1006
Largest diff. peak/hole /	5.63/-2.54	1.22/-1.21

e Å^{-3}
